# Transplantation of Patients with Hepatocellular Carcinoma Through Increased Utilization of Machine Perfusion Technology

**DOI:** 10.1097/TXD.0000000000001777

**Published:** 2025-03-10

**Authors:** Lauren E. Matevish, Jason Guo, Andrew D. Shubin, Malcolm MacConmara, Christine S. Hwang, Nathanael Raschzok, Nicole E. Rich, Arjmand R. Mufti, Amit G. Singal, Parsia A. Vagefi, Madhukar S. Patel

**Affiliations:** 1 Division of Surgical Transplantation, Department of Surgery, University of Texas Southwestern Medical Center, Dallas, TX.; 2 TransMedics, Andover, MA.; 3 Department of Surgery, Campus Charité Mitte, Campus Virchow-Klinikum, Charité – Universitätsmedizin Berlin, corporate member of Freie Universität Berlin and Humboldt-Universität zu Berlin, Berlin, Germany.; 4 Berlin Institute of Health at Charité – Universitätsmedizin Berlin, BIH Biomedical Innovation Academy, BIH Charité Clinician Scientist Program, Berlin, Germany.; 5 Division of Digestive and Liver Diseases, Department of Medicine, University of Texas Southwestern Medical Center, Dallas, TX.

## Abstract

**Background.:**

With the intent to mitigate waitlist disparities, the median model for end-stage liver disease (MELD) at transplant minus 3 policy nevertheless decreased access to liver transplant for patients with hepatocellular carcinoma (HCC). However, the adoption of machine perfusion (MP) technologies has shown promise in improving deceased donor graft yield and utilization. To understand current use for patients with HCC, we examined liver transplant patterns with MP and the characteristics of patients with HCC receiving an MP liver.

**Methods.:**

Adult patients with HCC undergoing deceased donor liver transplant from September 29, 2021, to March 30, 2024, were identified using the United Network for Organ Sharing Standard Transplant Analysis and Research files. Patients were excluded if listed as status 1A or they underwent multiorgan or split liver transplant. Multivariate analysis compared patients with HCC receiving an MP liver with those receiving a static cold storage liver.

**Results.:**

Of 3774 liver recipients with HCC, 593 (15.7%) underwent transplant with an MP graft. Compared with patients donation after circulatory death graft receiving a graft with static cold storage preservation, those with MP had less advanced disease (ie, Child-Pugh class C cirrhosis 22.9% versus 29.9%, *P* < 0.01) and lower median match MELD (13 versus 17, *P* < 0.001). Tumor characteristics were similar between groups, including alpha-fetoprotein level, maximum tumor size, and locoregional treatments. Donor factors, and not tumor burden, were most predictive of receipt of an MP liver (donation after circulatory death graft: odds ratio [OR], 14.81; macrosteatosis >30%; OR, 3.85; donor age older than 60 y; OR, 2.34). A shorter waitlist time (6.5 versus 7.2 mo, *P* < 0.01), with similar 1-y patient survival (93.6% versus 93.2%, *P* = 0.82) and graft survival (92.0% versus 91.6%, *P* = 0.84), was also noted in patients undergoing MP transplant.

**Conclusions.:**

The strategic use of MP livers may improve graft utilization and access to liver transplants, helping offset the disadvantages of the MELD at transplant minus 3 policy for patients with HCC.

Primary liver cancers, of which hepatocellular carcinoma (HCC) is the most common, are projected to affect >1 million individuals per year by 2025.^1^ Moreover, prognosis remains poor, with a 5-y survival of only ~20%.^1,2^ Transplantation is one of the few potentially curative treatments for patients with HCC, providing long-term survival rates as high as 70%.^3-7^ In the past decade, hepatobiliary malignancies have accounted for 20%–50% of liver transplants performed worldwide.^5,8-11^ However, both the shortage of deceased donor livers available for transplant and equity in allocating this scarce resource remain key challenges affecting waitlisted patients.

As the severity of their disease is often not adequately reflected in model for end-stage liver disease (MELD) scores, patients with HCC have historically been awarded MELD exception points to reflect their risk of death on the waitlist. In 2019, the Median MELD at Transplant minus 3 (MMAT-3) national policy was implemented to mitigate access and outcome disparities between patients with and without HCC in different transplant regions due to concern for overprioritization of patients with HCC. Although there have been noted improvements in non-HCC waitlist dropout rates, studies suggest MMAT-3 has resulted in decreased access to transplants for patients with HCC.^12,13^ Specifically, patients with HCC have a lower incidence of liver transplant^13^ and increased dropout rates,^12^ suggesting that MMAT-3 may have overcorrected, particularly after the implementation of the acuity circles policy.

Emerging technology in deceased donor organ salvage and preservation may be uniquely poised to increase the organ pool and address some of these concerns; indeed, the advent of ex vivo machine perfusion (MP) has revolutionized the field of organ transplantation. Although static cold storage (SCS) has been the gold standard for organ preservation for the past few decades, it is less effective for allografts of marginal quality.^14^ The progressive adoption of MP technologies has made the use of deceased donor grafts with higher-risk features (eg, donors after circulatory death, macrosteatotic or older grafts) more feasible. MP has been shown to reduce graft discard rates, prolong preservation time, and allow for viability testing, permitting graft salvage and increased organ utilization.^14-19^ Among other recipient benefits, it has helped mitigate ischemia/reperfusion injury (IRI) and liver allograft dysfunction.^20,21^

To better understand current practices, we sought to examine liver transplant patterns with MP in patients with HCC, as well as differences in patient and donor factors in recipients with HCC who received an MP liver compared with SCS.

## MATERIALS AND METHODS

The national United Network for Organ Sharing (UNOS) Standard Transplant Analysis and Research (STAR) files were queried to identify all adult patients with HCC undergoing deceased donor liver transplant from September 29, 2021, to March 30, 2024. These dates were chosen as the period following the US Federal Drug Administration approval of the first MP system. Patients were excluded if they were listed as status 1A, received a multiorgan transplant, or had a split liver. Subsequently, patients with HCC receiving an MP liver were compared with those receiving a liver preserved with SCS. Multiple types of MP are categorized within the STAR file database (normothermic, hypothermic, and other), and although normothermic MP (NMP) is the most common, all were included as part of the MP group.

The primary outcome was waitlist time, with secondary outcomes of 1-y patient and graft survival. Recipient variables included demographic data, Child-Pugh (CP) class, laboratory MELD score, match MELD, exception points, alpha-fetoprotein (AFP) level, number of tumors, largest tumor size, number of locoregional treatments (including transarterial chemoembolization, transarterial radioembolization, stereotactic body radiotherapy, and ablative therapies), as well as available explant pathology results (HCC presence, worst tumor differentiation, and vascular invasion). Donor variables included demographic data, donation after circulatory death (DCD) status, cause of death, terminal enzymes, graft steatosis, share type (local, regional, and national), and liver perfusion type. Laboratory MELD score was defined as the recipient’s final MELD score before to transplant, calculated as MELD-Na before July 2023 and MELD 3.0 after due to updates to the MELD formula. Match MELD was calculated as the recipient’s MELD score after exception point adjustments.

For univariate analyses, a comparison of continuous variables was done using the Mann-Whitney *U* test and Student *t* test as appropriate. The Pearson chi-square coefficient was applied to categorical and binary data elements. Median values with interquartile ranges were reported for nonparametric continuous variables, and mean values were reported for parametric continuous variables. A multivariable logistic regression model was performed to identify factors associated with receipt of an MP liver in patients with HCC. Variables were included if significant on univariate analyses (ie, *P* < 0.05), or deemed to be clinically relevant, that is, recipient age, sex, ethnicity, CP class, match MELD, time on waitlist, geographic region, donor age, DCD donor, share type, and presence of macrosteatosis of >30%.

As the most broadly adopted form of MP in the United States, we subsequently performed a subgroup analysis comparing waitlist times at NMP centers and non-user centers to better understand the existing effect that NMP access may have on patients with HCC. To adjust for timing of NMP initiation at centers, patients at NMP centers were included if they were activated on the waitlist on or after the date of the first NMP liver case at the listed center. At non-user centers, patients were included if they were listed on or after September 29, 2021. HCC wait times at NMP centers were compared with non-user centers and stratified by the MELD score. At NMP centers, the wait times of patients undergoing NMP transplants were subsequently compared with those who underwent non-NMP transplants.

To further assess the impact of MP utilization on waitlist outcomes, all liver transplant candidates registered after September 29, 2021, were stratified on the basis of listing center NMP utilization. Active centers were defined by an NMP utilization rate of at least 5% for all liver transplants from September 29, 2021, to March 31, 2024. A 5% cutoff was set to include only centers demonstrating NMP resource utilization, distinguishing them from centers that may have only used it once; using a percentage allows for normalizing for center volume and changes in volume over time. Patient waitlist outcomes at active NMP centers were compared with those at low/non-utilizing centers. A competing risk regression analysis using Fine-Gray models was performed to evaluate associations between center and recipient factors and patient-level time to LT by treating waitlist removal for death or deterioration and LT as competing risks. The effect of listing at an active NMP center was examined with the addition of clinically important variables, including candidate age, sex, and tumor factors.

A *P* value of < 0.05 was considered statistically significant. All analyses were performed using Stata version 18 (StataCorp, College Station, TX). This study was reviewed by the Institutional Review Board and determined to be exempt.

## RESULTS

A total of 3774 liver transplants were performed in recipients with HCC, of which 15.7% (N = 593) were preserved with MP; in comparison, only 11.7% of patients without HCC received an MP graft (*P* < 0.001). Of the MP livers transplanted into patients with HCC, 88.0% were preserved using NMP and 6.1% with hypothermic MP (HMP); 5.9% were classified as “other.”

There were few significant differences in demographics between patients with HCC receiving an MP versus SCS graft (Table 1). Patients were predominantly men (75.9% versus 73.5%, *P* = 0.23), with an average age of 62.6 versus 61.9 y (*P* = 0.07). Additionally, most recipients were White (63.4% versus 62.2%), followed by Hispanic (23.4% versus 22.8%) and Asian (6.6% versus 7.5%). Patients with HCC undergoing LT with MP were less likely to have CP class C cirrhosis (22.9% versus 29.3%, *P* < 0.01) and had both a lower median laboratory MELD (13 [9–18] versus 13 [9–21], *P* = 0.01) and median match MELD (13 versus 17, *P* < 0.001) at transplant than those with SCS preservation. A similar number of patients in each group had active exception points (80.3% versus 80.9%; *P* = 0.74). HCC tumor factors were relatively matched between groups, including median AFP level, median number of tumors, largest tumor size, total number of locoregional treatments, and prior surgical resection (Table 1). On explant pathology, nearly half of tumors were moderately differentiated (47.7% versus 45.1%), and another quarter had total tumor necrosis (25.8% versus 29.4%). Only a small percentage in both groups had micro- or macrovascular invasion (10.4% versus 11.2%).

**TABLE 1.** Comparison of recipient characteristics in patients with HCC undergoing LT with MP vs SCS

	LT with MP (N = 593)	LT with SCS (N = 3181)	*P*
Age, y, mean ± SD	62.6 ± 8.6	61.9 ± 9.1	0.07
Sex (male)	75.9%	73.5%	0.23
Ethnicity			0.93
White	376 (63.4%)	1978 (62.2%)	
Black	31 (5.2%)	191 (6.0%)	
Hispanic	139 (23.4%)	725 (22.8%)	
Asian	39 (6.6%)	240 (7.5%)	
BMI, kg/m^2^, ±SD	29.3 ± 5.3	29.3 ± 5.6	0.99
Child-Pugh class			<0.01
A	181 (30.5%)	889 (27.9%)	
B	276 (46.5%)	1342 (42.2%)	
C	136 (22.9%)	950 (29.9%)	
Laboratory MELD, median (IQR)	13 (9–18)	13 (9–21)	0.01
Match MELD, median (IQR)	13 (9–19)	17 (10–27)	<0.001
Active exception points	80.3%	80.9%	0.74
AFP level, median (IQR)	5.5 (3–11)	5 (3–11)	0.98
No. of tumors, median (IQR)	1 (1–1)	1 (1–1)	<0.01
Maximum tumor diameter, median (IQR)	3.0 (1.9–4.3)	3.0 (1.9–4.5)	0.86
Total locoregional treatments, median (IQR)	1 (1–2)	1 (1–2)	0.37
Pre-LT surgical resection	2.5%	2.1%	0.57
Worst tumor differentiation			0.37
Total tumor necrosis	72 (25.8%)	495 (29.4%)	
Well	61 (21.9%)	325 (19.3%)	
Moderate	133 (47.7%)	758 (45.1%)	
Poor	13 (4.7%)	104 (6.2%)	
Vascular invasion			0.19
None	250 (89.6%)	1493 (88.8%)	
Microvascular	28 (10.0%)	158 (9.4%)	
Macrovascular	1 (0.4%)	31 (1.8%)	
Months on waitlist, median (IQR)	6.5 (1.6–10.4)	7.2 (2.5–11.3)	<0.01
1-y graft survival	92.0%	91.6%	0.84
1-y patient survival	93.6%	93.2%	0.82

AFP, alpha-fetoprotein; BMI, body mass index; HCC, hepatocellular carcinoma; IQR, interquartile range; LT, liver transplant; MELD, model for end-stage liver disease; MP, machine perfusion; SCS, static cold storage.

Donor factors differed between groups on several key variables (Table 2). MP donors were older (older than 40 y, 72.2% versus 61.9%, *P* < 0.001) and more likely to have macrosteatosis of >30% (3.9% versus 1.6%, *P* < 0.001). Grafts preserved with MP were also overwhelmingly from DCD donors (61.0% versus 13.6%, *P* < 0.001) with a slightly higher incidence traumatic deaths (52.4% versus 47.6%, *P* = 0.2). MP and SCS groups did not differ in donor sex (men 63.1% versus 62.0%, *P* = 0.62), body mass index (29.2 versus 28.9, *P* = 0.39), or rates of high terminal enzymes (AST >500; 1.7% versus 1.2%, *P* = 0.39).

**TABLE 2.** Comparison of donor factors in patients with hepatocellular carcinoma undergoing LT with MP vs SCS

	LT with MP (N = 593)	LT with SCS (N = 3181)	*P*
Age, y			<0.001
<40	165 (27.8%)	1211 (38.1%)	
40–60	272 (45.9%)	1174 (36.9%)	
>60	156 (26.3%)	796 (25.0%)	
Sex (male)	63.1%	62.0%	0.62
BMI, kg/m^2^, mean ± SD	29.2 ± 7.3	28.9 ± 7.1	0.39
Cause of death			0.031
Trauma	297 (52.4%)	1481 (47.6%)	
Anoxia	168 (29.6%)	926 (29.8%)	
CVA	102 (18.0%)	703 (22.6%)	
DCD %	61.0%	13.6%	<0.001
Liver perfusion type			
Normothermic	522 (88.0%)	NA	
Hypothermic	36 (6.1%)	NA	
Other	35 (5.9%)	NA	
Share type			0.09
Local	253 (42.7%)	1449 (45.6%)	
Regional	183 (30.9%)	844 (26.5%)	
National	157 (26.5%)	888 (27.9%)	
Enzymes (AST >500)	1.9%	1.2%	0.19
Macrosteatosis (>30%)	3.9%	1.6%	<0.001

AST, aspartate transaminase; BMI, body mass index; CVA, cerebrovascular accident; DCD, donor after circulatory death; LT, liver transplant; MP, machine perfusion; SCS, static cold storage.

Regional variation in practice patterns was subsequently examined by looking at the share of MP grafts transplanted into recipients with HCC compared with total MP grafts transplanted in each Organ Procurement and Transplantation Network region. Regions 2 (20/69; 29.0%), 5 (201/821; 24.5%), and 7 (25/104; 24.0%) had the highest rates, whereas regions 9 (14/97; 14.4%), 3 (45/283; 15.9%), and 11 (54/324; 16.7%) demonstrated the lowest (Table 3). By absolute numbers, regions 5 (N = 201) and 4 (N = 86) transplanted the most patients with HCC with MP livers; regions 2, 6, 7, and 9 each transplanted <25 MP livers into patients with HCC. Differential use in MP grafts for patients with HCC and patients without HCC was evaluated by comparing the regional rate of MP use for patients with HCC with the regional rate of MP use for patients without HCC. Regions 2 (2.38), 7 (1.82), and 5 (1.54) all had ratios >1.5, denoting high rates of MP graft use in patients with HCC compared with patients without HCC; region 9 was the only area with a ratio under 1, indicating a lower likelihood of using MP grafts for patients with HCC.

**TABLE 3.** Number of MP livers transplanted into patients with and without HCC and comparison of differential perfusion rates in patients with HCC and without HCC stratified by OPTN region

OPTN region	Patients with HCC (N = 593)	Patients without HCC (N = 2332)	% MP grafts going to HCC recipients	MP differential use ratio^*a*^
1	56	201	21.8%	1.46
2	20	49	29.0%	2.38
3	45	238	15.9%	1.19
4	86	375	18.7%	1.15
5	201	620	24.5%	1.54
6	9	38	19.1%	1.21
7	25	79	24.0%	1.82
8	31	138	18.3%	1.25
9	14	83	14.4%	0.83
10	52	241	17.7%	1.41
11	54	270	16.7%	1.20

^*a*^MP differential use ratio defined as (% of patients with HCC receiving an MP graft)/(% of patients without HCC receiving an MP graft).

HCC, hepatocellular carcinoma; MP, machine perfusion; OPTN, Organ Procurement and Transplantation Network.

Independent factors associated with receipt of a machine-perfused liver in patients with HCC included several recipient and donor predictors (Table 4). Match MELD was slightly but significantly inversely associated with receipt of an MP liver (adjusted odds ratio [aOR], 0.94; 95% confidence interval [CI], 0.92-0.96). No HCC tumor factors were significantly associated with undergoing LT with an MP liver. Predictive donor factors included older donor age (>60 y; aOR, 2.34; 95% CI, 1.61-3.40), macrosteatosis >30% (aOR, 3.85; 95% CI, 1.72-8.63), and most strikingly, DCD donation (aOR, 14.81; 95% CI, 10.49-20.91). Geographically, patients with HCC in the West (regions 5 and 6) were more likely to undergo MP transplant (aOR, 3.04; 95% CI, 11.91-4.86 versus Northeast reference).

**TABLE 4.** Factors associated with receipt of a machine-perfused liver in patients with hepatocellular carcinoma on multivariable logistic regression

Factors	Odds ratio (95% CI)	*P*
Recipient age, y	1.02 (1.00-1.04)	0.09
Recipient sex (male)	1.29 (0.90-1.83)	0.16
Ethnicity		
White	Reference	
Black	1.36 (0.74-2.50)	0.32
Hispanic	1.12 (0.77-1.62)	0.55
Asian	0.67 (0.37-1.20	0.18
Match MELD	0.94 (0.92-0.96)	<0.001
AFP level	1.00 (1.00-1.00)	0.25
No. of tumors	0.87 (0.64-1.18)	0.37
Maximum tumor diameter	0.95 (0.88-1.02)	0.14
Total locoregional treatments	0.99 (0.89-1.09)	0.80
Months on waitlist	1.00 (1.00-1.00)	<0.01
DCD donor	14.81 (10.49-20.91)	<0.001
Donor age (>60 y)	2.34 (1.61-3.40)	<0.001
Enzymes (AST >500)	2.00 (0.65-6.40)	0.24
Macrosteatosis (>30%)	3.85 (1.72-8.63)	0.001
Share type		
Local	Reference	
Regional	1.81 (1.28-2.55)	0.001
National	1.73 (1.16-2.58)	<0.01
Region		
Northeast	Reference	
Midwest	0.79 (0.47-1.33)	0.38
South	1.54 (0.97-2.42)	0.07
West	3.04 (1.91-4.86)	<0.001

AFP, alpha-fetoprotein; AST, aspartate transaminase; CI, confidence interval; DCD, donor after circulatory death; LT, liver transplant; MELD, model for end-stage liver disease.

**FIGURE 1.**
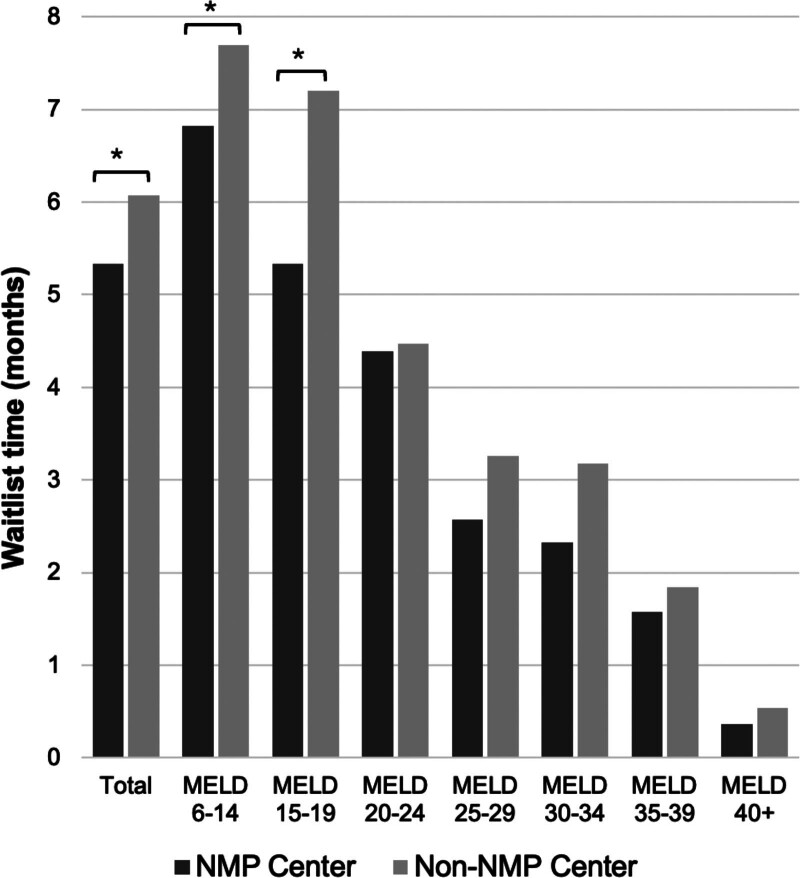
Mean waitlist times for patients with HCC listed at centers utilizing NMP vs those not utilizing NMP, stratified by laboratory MELD score. HCC, hepatocellular carcinoma; MELD, model for end-stage liver disease; NMP, normothermic machine perfusion.

Clinical outcomes were similar among patients with HCC who received an MP or SCS graft, including no significant differences in 1-y graft survival (92.0% versus 91.6%, *P* = 0.84), or 1-y patient survival (93.6% versus 93.2%, *P* = 0.82; Table 1). However, median waitlist times were shorter for patients undergoing MP transplants, at a median of 6.5 mo compared with 7.2 mo (*P* < 0.01). On subanalysis, overall mean waitlist times for patients with HCC at NMP-utilizing centers were significantly shorter than at non-NMP centers (5.3 versus 6.1 mo, *P* = 0.0016; Figure 1). This impact was most notable at low MELD scores. Patients with a laboratory MELD score of 6–14 had an average waitlist time of 6.8 mo at NMP centers and 7.7 mo at non-NMP centers (*P* = 0.0051); at a laboratory MELD score of 15–19, the difference was 5.3–7.2 mo (*P* = 0.0014). No difference was seen between user and non-user centers in HCC patients with a MELD score of ≥20.

Waitlist outcomes for candidates listed at active NMP centers were compared with low or non-utilizing centers. A slightly higher percentage of patients listed at active NMP centers underwent LT during the study period (64.1% versus 60.7%), with fewer removals due to deterioration (11.6% versus 12.4%; Table 5). On the competing risk regression model, the incidence of liver transplant when listed at an active NMP center was associated with a subdistribution hazard ratio (sHR) of 1.16 (95% CI, 1.05-1.27; Table 6). The total number of locoregional HCC treatments was also significant within the model, with a lower sHR of liver transplant with an increasing number of treatments (sHR, 0.84; 95% CI, 0.82-0.88).

**TABLE 5.** LT waitlist outcomes for candidates registered at centers with active use of NMP compared with those at low or non-utilizing centers

	Patients listed at active NMP centers (N = 1906)	Patients listed at non-utilizing centers (N = 2248)	
Still listed	134 (7.0%)	199 (8.9%)	<0.001
Underwent LT	1157 (60.7%)	1442 (64.1%)	
Deteriorated	237 (12.4%)	260 (11.6%)	
Improved	48 (2.5%)	36 (1.6%)	
Death	86 (4.5%)	100 (4.4%)	
Died during LT	1 (0.1%)	2 (0.1%)	
Other	243 (12.7%)	209 (9.3%)	

LT, liver transplant; NMP, normothermic machine perfusion.

**TABLE 6.** Multivariate competing risks regression analysis for liver transplantation among waitlisted candidates with hepatocellular carcinoma, with waitlist removal for death or deterioration as competing risks

	sHR (95% CI)	*P*
Active NMP center^*a*^	1.16 (1.05-1.27)	0.002
Candidate age, y	0.99 (0.98-1.00)	0.001
Candidate sex (male)	1.01 (0.91-1.13)	0.82
Laboratory MELD	0.99 (0.98-1.00)	0.003
AFP level	1.00 (1.00-1.00)	0.004
Number of tumors	1.00 (0.91-1.09)	0.99
Maximum tumor size	0.97 (0.94-1.00)	0.04
Total locoregional treatments	0.84 (0.82-0.88)	<0.001

^*a*^Active NMP centers defined by the utilization of NMP in at least 5% of all center liver transplants from September 29, 2021, to March 31, 2024.

AFP, alpha-fetoprotein; CI, confidence interval; ; MELD, model for end-stage liver disease; NMP, normothermic perfusion; sHR, subdistribution hazard ratio.

## DISCUSSION

Although MP implementation is relatively nascent and indications for use are evolving, early data from the UNOS national database demonstrate that MP livers are more often transplanted into patients with HCC than other indications. In the subpopulation of transplanted patients with HCC, MP livers had more marginal qualities than their SCS counterparts and were transplanted into recipients with lower MELD and CP scores. Notably, there was a small but significant difference in wait times between MP and SCS groups, with similar 1-y graft and patient survival. For patients with a MELD score of <20, those listed at NMP-utilizing waited nearly 2 mo less in some cases. The clinical relevance of this additional wait time likely hinges on patient, treatment, and tumor-level factors, which may come to light as the field evolves. We extrapolate that marginal and extended criteria livers that are salvaged through the use of MP technology are providing viable livers to patients who may otherwise have a prolonged wait due to their allocation priority.

Indeed, since the enactment of the MMAT-3 policy, several studies have suggested that patients with HCC are now disadvantaged in their access to transplants. Bernards et al^12^ found that although this policy change resulted in a 10% decreased overall risk of waitlist dropout for patients without HCC, patients with HCC were 57% more likely to drop out than listees without HCC on adjusted analysis. Also, problematic within MMAT-3 is its disregard for disease severity; patients with HCC residing in a certain catchment area are listed with the same priority despite differences in tumor size, treatment response, and liver function, which inherently confer different mortality risks. The acuity circle policy, enacted in February 2020, has additionally exacerbated these concerns, with regional reductions in HCC transplants.^13,22,23^ We found no major differences between HCC tumor factors between groups, and more intentional use of MP for patients at high risk of waitlist dropout could help to facilitate transplant. Further iterations to allocation policy should aim to address these concerns.

Prior Organ Procurement and Transplantation Network studies demonstrated that the donation service area in which a candidate was waitlisted was most highly associated with disparities in transplant access^24,25^; although donation service areas are no longer used for distribution, geographic inequities persist. Our data show that transplantation in the West was independently associated with the receipt of an MP graft on multivariable analysis. However, this is likely not driven by the region but rather by a few high-volume, high-utilizer centers with the capacity to absorb the financial and human resource burden of MP. The subanalysis of waitlist times and outcomes at active NMP centers gives further evidence of widening gaps in access. As such, these programs may be better poised to provide timely transplants to their waitlisted candidates with HCC. Given the relative infancy of perfusion adoption in the United States and the high associated costs, mitigating inequities as perfusion technologies are implemented remains crucial.

In addition to expanding the organ pool and providing patients access to allografts, transplantation with ex vivo MP may be particularly desirable for the HCC cohort, as IRI and higher-risk donor features are associated as risk factors for tumor recurrence.^26-29^ Several studies have demonstrated a clinical and biochemical protective effect of MP against reperfusion syndrome,^17,30,31^ and in animal models, mediation of inflammatory cascades leading to a reduction in IRI has shown to decrease HCC recurrence.^32^ It is suggested that through similar mechanisms, MP is able to reduce tumor recurrence, even when including patients outside traditional Milan criteria.^33^ Although current estimates of tumor recurrence after transplant are around 15%,^3^ Mueller et al^33^ reported 5-y rates of tumor-free survival at 92% utilizing HMP. Other studies have noted equivalent, if not reduced, rates of recurrence using HMP compared with SCS.^34^ Notably, this association has yet to be well studied with NMP due to limited available follow-up to date. More robust work needs to be done in this space to assess the long-term benefits of all types of MP, specifically for its use in HCC treatment.

There are limitations within the data set and analysis performed that warrant mention. The decision to use MP for a liver allograft is based on many donor, recipient, and program-level factors, some of which may not be well captured within the UNOS database (eg, operating room availability, multiple concurrent liver transplants, concern for prolonged hepatectomy phase). In addition, HMP cases are included within the MP subset, although most of these grafts are preserved as part of clinical trials, in which decision-making can differ from real-world application. In the context of MP, updates to the UNOS forms should be considered for improved data collection, particularly in the documentation of cold ischemia time (currently includes time on pump) and specific perfusion technology designations (eg, clarifying the current nonspecific “other” category, and creation of an option or unique field for normothermic regional perfusion). For example, the current UNOS STAR files MP documentation does not distinguish between “back-to-base” and “up-front” NMP models or the use of multiple technologies (eg, normothermic regional perfusion followed by MP). This may result in an underestimation of the true number of MP cases within the data set in its current form. The incompleteness of the data set regarding HCC variables is an additional limitation, with about one-half missing explant pathology and 19% missing granular treatment and exception data. Notably, accounting for the full impact of MP in the MMAT-3 era is difficult due to the additional confounding contributed by the acuity circles policy, all amidst a background of the COVID-19 pandemic. Perhaps the most important limitation to keep in mind is the novelty of MP technology in such that long-term follow-up data are still lacking, including data about HCC recurrence.

Despite the limitations noted before, this study is the first to assess the use of MP technologies, specifically in patients with HCC using a large national database, and how NMP-utilizing centers have been able to minimize waitlist times and increase the probability of transplantation. Although gaps in the current tumor-specific data within the STAR files database preclude a more robust analysis, further investigation into the current HCC exception point policy is warranted, as it fails to address the heterogeneity of waitlist dropout and recurrence in the population of patients with HCC. Still, based on the available data presented here, patients with HCC with lower MELD scores may be optimal recipients of traditionally higher-risk grafts preserved with MP, improving their access to liver transplantation given recent policy changes portending longer wait times.
